# The cutaneous microbiome: microbial remodeling of the skin lipid landscape

**DOI:** 10.1128/msphere.00648-25

**Published:** 2026-06-10

**Authors:** Sylvia B. Khalil, Caitlin H. Kowalski

**Affiliations:** 1 Department of Dermatology, Dartmouth Hitchcock Medical Center 22916, Lebanon, New Hampshire, USA; 2 Department of Dermatology, Geisel School of Medicine 849109, Hanover, New Hampshire, USA; 3 Department of Microbiology and Immunology, Geisel School of Medicine 12285 , Hanover, New Hampshire, USA; Shenzhen Institute of Synthetic Biology, Chinese Academy of Sciences, Shenzhen, China

**Keywords:** skin microbiome, host-pathogen interactions, skin lipids, skin microbiota, fungal-bacterial interactions

## Abstract

The human skin microbiome exists within a lipid-rich environment that profoundly shapes microbial colonization, metabolism, and interactions with the host. Resident microbes not only consume host-derived lipids for energy but also transform them into bioactive compounds that influence microbial competition, immune responses, and skin barrier function. In turn, microbially generated lipids become part of the skin lipid landscape, including short-chain fatty acids and cell envelope lipids, and contribute to maintaining homeostasis or, in some cases, influence disease processes such as acne and atopic dermatitis. Despite increased recognition of their importance, the diversity, origins, and bioactivities of microbially derived lipids on skin remain underexplored. Here, we highlight recent advances in understanding how cutaneous microbes shape and are shaped by the skin lipid landscape, emphasizing the potential of microbial lipids as mediators of skin health, disease, and novel therapeutic strategies.

## INTRODUCTION

Skin serves the essential function of protecting internal tissues. Through multiple layers of tightly packed cells held together by proteins and lipids, stabilizing connective tissue, and a deep layer of adipose tissue, the skin forms a barrier against mechanical damage from the environment while also preventing potentially harmful microbes and toxins from entering the body. This physical barrier relies on the differentiation of epidermal keratinocytes into dead, flattened, anucleate corneocytes in a process referred to as cornification. This process establishes the stratum corneum, the outermost layer of the epidermis, where the corneocytes are embedded within a lipid matrix, generating a selectively permeable barrier (1). In combination with this physical layer, the skin’s chemical defense system is composed of antimicrobial peptides and lipids that respond to and modulate the microenvironment. These secreted products can directly prevent the colonization, invasion, or expansion of pathogenic microbes (2–4), while also inducing an immune response within the skin as a defensive strategy (5–7). The skin’s other functions, which include sensing the surrounding environment, regulating body temperature, and preventing dehydration, serve similarly critical roles in the protection of the body from the external world.

In its role as a protective barrier, skin does not act alone. Augmented by the skin microbiome, a community of bacteria, viruses, fungi, and microeukaryotes that live on and within the skin, the protective function of the skin is enhanced. These skin resident microbes produce antimicrobials (8–11), compete with neighboring and undesirable microbes for nutrients (12, 13) (a phenomenon better described in the gut [14]), and prime the body’s defenses through immune learning (15, 16). Notably, these microbes are not only present on the epidermis but also span to deeper layers of the skin, residing in protected niches like hair follicles (17, 18). Thus, the use of deeper, more invasive sampling techniques may be necessary to fully characterize all inhabitants of the skin microbiome (17–20).

The skin of adults is highly heterogeneous and contains several unique environments, each providing a favorable site for different microbes to colonize. Some of the most abundant bacterial genera on the skin are *Cutibacterium* (formerly known as *Propionibacterium*), *Corynebacterium*, and *Staphylococcus* (21–27). Sebaceous, or oily, sites (such as the forehead and side of the nose) mainly contain *Cutibacterium* and *Staphylococcus* spp., while moist sites (such as the armpit or inner elbow) are dominated by corynebacteria and some staphylococci. The dry mid forearm and buttock sites have the greatest diversity, with notable colonization by gram-negative bacteria (24–26). Analysis of skin viral communities, particularly those with DNA genomes, has revealed a dominance of bacteriophages that mirror the spatial patterns of their skin-resident bacterial hosts, with eukaryotic viruses represented by papillomaviruses, herpesviruses, and polyomaviruses also present (24, 28). While bacterial commensals and their phages vary based on region, the fungal community composition is consistent across most body sites except the feet. This fungal component, called the mycobiome, is dominated by the singular genus *Malassezia,* with minor contributions by *Candida, Rhodotorula,* and several other environmental yeasts and molds (27). The microscopic arthropod mites, *Demodex folliculorum* and *Demodex brevis,* are present on healthy adult skin and localize to sebaceous sites, specifically the face (29). Despite this diversity, most investigations of the skin microbiome have focused on skin-resident bacteria.

Recently, investigators have used propidium monoazide (PMA)-based 16S rDNA amplicon sequencing to distinguish viable from non-viable bacteria on the skin. PMA does not penetrate viable cells, but readily enters dead cells and binds DNA, also called relic DNA. PMA-DNA crosslinking prevents downstream molecular analysis (e.g., PCR amplification, library preparation), thereby enriching samples for DNA from viable cells in subsequent DNA sequencing. These studies suggest that bacterial richness and diversity within the stratum corneum are lower than previously estimated, with most viable bacteria localized to protected skin structures such as hair follicles and other invaginations (30). A subsequent PMA-based shotgun metagenomic study confirmed a reduced abundance of viable bacterial cells from the epidermis compared to conventional methods but did not observe significant differences in bacterial diversity or evenness (31). Notably, comparable analyses of viable versus non-viable fungal communities have not yet been conducted; however, other studies suggest that *Malassezia* resides within the upper regions of hair follicle structures (20, 32).

While PMA-based methods to differentiate viable from non-viable microbes have transformed our understanding of microbial abundance on human skin, these approaches are not without limitations (33). The ability of PMA to accurately differentiate between viable and non-viable DNA can be influenced by several factors, including the sample type, community composition, and microbial biomass, which may underlie the different results (33). As such, complementary methods, such as skin microbiome metatranscriptomics, to evaluate microbial viability via transcriptional activity are needed. To date, we are aware of only two studies that have investigated transcriptomes of the human skin microbiome (34, 35). In the most recent study, Chia et al. used skin swabs from anatomically diverse skin sites and observed clear differences between transcriptional and genomic abundances, without the use of PMA (35). While this does not indicate microbial viability, it suggests that the most transcriptionally active microbes on the skin are not necessarily the most abundant. Notably, while PMA-based studies have yet to be applied to the skin mycobiome, metatranscriptomics data suggest that *Malassezia* species are viable and transcriptionally active on human skin. In fact, although *Malassezia* constitutes a small fraction of overall microbial abundance based on metagenomics, *Malassezia globosa* and *Malassezia restricta* transcripts represent large components of overall microbial transcriptomes at most skin sites (35). This suggests that *Malassezia* are viable and transcriptionally active at multiple human skin sites.

Sebaceous sites represent a distinct and selective niche for the skin microbiome, characterized by lower microbial diversity, evenness, and richness compared to other skin environments (25). For microbes present across multiple skin sites, such as *Staphylococcus epidermidis* and *Cutibacterium acnes*, metatranscriptomics data indicate that transcriptional activity is the highest in sebaceous regions (35). These sites contain a high density of hair follicles and sebaceous glands (36), which together form the pilosebaceous unit, a structure composed of a hair follicle and its associated sebaceous gland, establishing a unique microbial habitat (37). Sebum, the substance produced by sebaceous glands, contains a variety of lipids that serve as nutrients for some microbes while exerting antimicrobial effects on others (38). In addition, since the majority of viable bacteria are not found on the epidermal surface but rather within hair follicles, these appendages may serve as microbial reservoirs for skin recolonization by commensal skin microbes following epidermal perturbation (30). The selective yet hospitable nature of this environment may be driven by the higher pH, increased moisture, reduced UV exposure, and distinct nutrient availability within the pilosebaceous unit (39). Collectively, these appendages substantially expand the functional surface area available for host-microbe interactions, increasing it from approximately 2 m^2^ on the epidermal surface to an estimated 25 m^2^ when skin appendages are considered (40).

Sebum production increases dramatically during puberty, remains relatively stable throughout most of adulthood, and declines in older age (41). Despite constant exposure to the external environment, commensal species within sebaceous sites colonize stably over time in healthy adults when sebum production is stable (42). Prior to this stability, the surge in sebum production during puberty coincides with pronounced shifts in skin microbial community composition, including enrichment of lipophilic taxa such as *Malassezia* species and *C. acnes* (43). Conversely, reduced sebaceous gland area and sebum production in older individuals correlates with a decline in lipophilic bacteria, such as *Cutibacterium* (23, 44, 45). This pattern aligns with reports of age-associated decreases in sebum levels in women (22, 23, 45), but not in men (46), likely reflecting the changes in androgen production, the hormone that stimulates sebaceous activity, following menopause (41). While other age-related changes in skin physiology and microbial composition have been described, it remains unclear what role the skin microbiome plays in shaping these changes in response to the altered lipid environment (44). These findings underscore that when sebaceous gland activity is stable, as during adulthood, the microbiome within these niches is similarly stable, whereas shifts in sebum production, and thus the skin lipid landscape, can dramatically reshape the microbial community structure.

**Fig 1:**
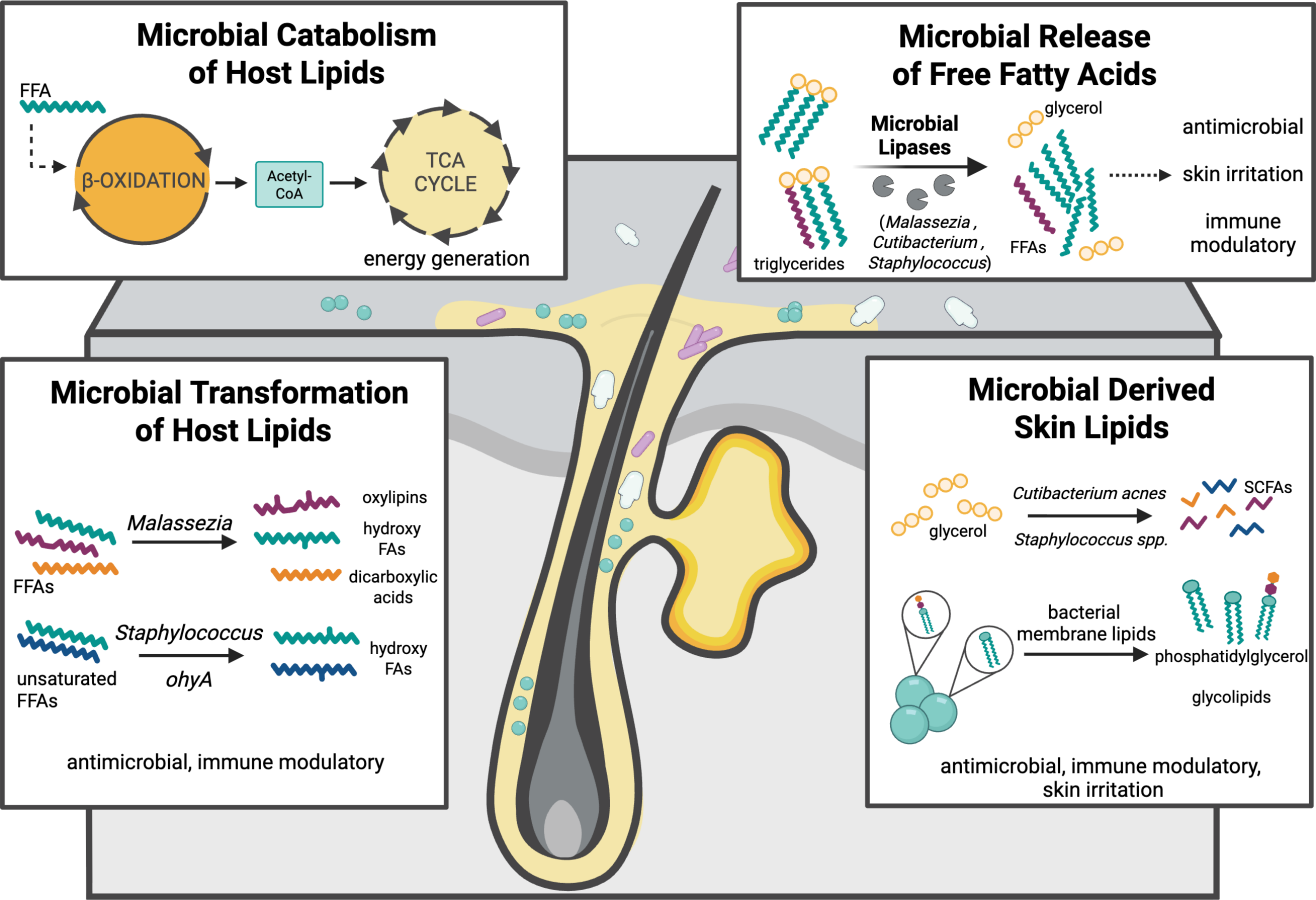
Microbial remodeling of the skin lipid landscape. Skin resident microbes catabolize skin lipids for energy, and in doing so, they release bioactive free fatty acids (FFAs) into the skin milieu. These FFAs can be further transformed by the microbes, generating secondary bioactive lipid metabolites that impact the host and neighboring microbes. Lipids derived from microbial *de novo* metabolism also become part of the skin lipid landscape and are poised to impact the host and microbiome. This image was created in BioRender.

Together, these observations position sebaceous glands and the skin lipidome as central organizers of the skin microbiome. Beyond shaping which microbes can persist on the skin, the environment created by sebaceous and epidermal lipids provides substrates for extensive microbial metabolism, generating a diverse array of bioactive lipid molecules. In the sections that follow, we examine how skin-associated microbes access, modify, and produce lipids, and how these microbially derived lipids influence microbial interactions, skin barrier function, and host immune responses. By framing the skin as a lipid-driven ecosystem, this review highlights the skin microbiome not as a passive inhabitant of the cutaneous surface, but as an active metabolic partner in shaping skin physiology and health (Fig. 1).

## SKIN LIPIDS PROVIDE A RICH ENERGY SOURCE FOR RESIDENT MICROBES

The skin habitat is acidic, desiccated, and considered a relatively nutrient-poor environment for microbial colonization (47). Despite these constraints, the skin is enriched in a diverse array of lipids that provide a rich energy source for microbes capable of exploiting them, selecting for the colonization of lipophilic bacteria and fungi. A recent study systematically examining the ability of diverse skin bacteria to grow in lipid-rich conditions revealed substantial inter- and intra-species variability in lipid preference, with several *Corynebacterium* and *Dietzia* strains displaying strong lipophilic growth phenotypes (38). The authors also identified a skin isolate of *Corynebacterium kefirresidentii* that was completely unable to grow in the absence of lipids. It is well documented that *Malassezia* yeasts and some *Corynebacterium,* such as *C. jeikeium,* lack the capacity for *de novo* fatty acid synthesis and rely entirely on exogenous lipid sources for growth (48, 49). The discovery of the *C. kefirresidentii* lipid dependency suggests that the prevalence of lipid-dependent bacteria within the skin microbiota may be underestimated.

Lipophilic members of the skin microbiota can access lipids derived from both the stratum corneum and sebaceous gland secretions. Epidermal lamellar membranes, which are formed during keratinocyte cornification, are essential for maintaining the water-tight barrier of the skin and function as the lipid “glue” between terminally differentiated corneocytes. During this process of keratinocyte differentiation, lipid-laden lamellar bodies are secreted along with hydrolytic enzymes that modify their lipid cargo. These enzymes process phospholipids and sphingolipids to generate free fatty acids (FFAs), ceramides, and sphingosine (50). Resident microbes can metabolize these liberated FFAs through β-oxidation, incorporate them into membrane phospholipids, or deploy their own lipases and hydrolases to further degrade more complex lipid species (51–55). Alterations in epidermal lipid composition, such as changes in the acyl chain length of lamellar ceramides, have been shown to correlate with shifts in skin microbial community structure (56). For example, in a murine model, increased epidermal FFA levels resulting from a high-fat diet are associated with elevated abundances of *Corynebacterium* species (56, 57). Together, these findings support a model whereby epidermal lipids are a key nutrient source for cutaneous microbes.

Sebum, produced by sebaceous glands, represents an additional and abundant lipid source for the skin microbiota. Sebum is produced by special secretory cells called sebocytes via holocrine secretion within sebaceous glands, which can occur at densities of up to approximately 900 glands per cm² in sebaceous-rich regions of human skin, such as the forehead and scalp (58). These glands can occur in isolation or be associated with hair follicles, and sebum secretion is critical for maintaining skin and hair lubrication, limiting transepidermal water loss (TEWL), maintaining thermoregulation, and contributing to defense against pathogens (50, 59). Chemically, sebum is composed of cholesterol; squalene; wax esters; tri-, di-, and mono-glycerides; and FFAs (38, 60). On the human body, wax esters are largely unique to sebum, where they account for roughly 25% of sebum lipids (61). While some microbes possess the ability to metabolize wax esters, it is unclear how the skin microbiome may utilize this unique lipid source (62). Sebum also contains unique FFAs not usually found elsewhere in the human body, including sapienic acid (C16:1, ∆6) and its elongated derivative sebaleic acid (C18:2, ∆5,8) (61). The precise composition of sebum varies between individuals, is influenced by factors such as diet (57, 63) and hormones (64), and can even reflect other changes in overall host health (65). While it is not possible to capture the precise and highly variable compositions of human sebum in the laboratory, synthetic sebum formulations have become widely used to model sebaceous environments *in vitro*. These formulations provide tractable systems for interrogating the role of these skin lipids in microbial physiology and ecological interactions (38, 66). Collectively, the specialized lipid metabolism required for microbial persistence on skin positions the skin microbiota not only as consumers of host lipids but also as active modifiers of the cutaneous lipid landscape.

## MICROBIOTA RELEASE OF BIOACTIVE FATTY ACIDS FROM SKIN LIPIDS

One of the most direct ways in which the skin microbiota reshapes the cutaneous lipid landscape is through the hydrolysis of host-derived lipids into FFAs. It has long been recognized that *C. acnes*, which resides primarily within hair follicles, expresses lipases that hydrolyze sebum triglycerides, resulting in the release of FFAs (67–70). Similarly, *Malassezia* species release FFAs from triglycerides when grown on sebum-based media *in vitro*, reflecting their reliance on exogenous lipids for growth (71). Triglycerides are not the only skin lipids to be degraded by the microbiota, as a recent study identified a sphingomyelinase produced by *S. epidermidis* capable of hydrolyzing epidermal sphingomyelin and thereby releasing ceramides and phosphocholine (72). While there are likely more examples of microbial degradation of complex skin lipids, in this section, we will focus on the generation of FFAs and their bioactivity.

Analysis of human facial skin estimates that 95% of FFAs on the skin surface are derived from sebum lipids, compared to 5% from stratum corneum lipids, however, freshly secreted sebum has low FFA content (73). This suggests that microbial metabolism plays a significant role in the release of FFAs from sebum triglycerides. Although the main purpose of this lipolytic activity is likely to generate glycerol and FFAs for microbial consumption, the breakdown of lipids can have broader implications when the released FFAs exhibit bioactive properties. The most abundant FFAs in human sebum, as a result of host and microbial lipid hydrolysis, are palmitic acid (C16:0), sapienic acid (C16:1, ∆6), stearic acid (C18:0), myristic acid (C14:0), and oleic acid (C18:1, ∆9) (74). In the context of this review, we define a bioactive FFA (or bioactive lipid) as a lipid molecule that, upon release or modification, exerts an observed biological effect on microbial or host cells independent of its role as a metabolic substrate. Such effects may include antimicrobial activity, modulation of host cell signaling, or alteration of skin barrier function.

Released FFAs can directly inhibit microbial growth, an action that can serve to shape microbial community composition and limit colonization by invading pathogens. For example, the lipophilic skin commensal *Corynebacterium accolens* releases FFAs that inhibit the growth of pathogenic *Streptococcus pneumoniae* (75). Hydrolysis of sebum lipids to generate the human-specific monounsaturated FFA, sapienic acid, is also thought to contribute to the skin’s intrinsic antimicrobial defense, including the inhibition of *Staphylococcus aureus* (76). Beyond direct antimicrobial effects, FFAs released by microbial lipolysis contribute to the acidic pH of the skin surface and can integrate into the stratum corneum of the epidermis, thereby strengthening the skin barrier and indirectly limiting pathogen colonization (77). The release of FFAs may also support the growth of some skin residents. The skin resident yeast *Malassezia restricta* cannot grow in the absence of lipids but grows more robustly in the presence of FFA compared to triglycerides (73). This suggests that FFAs released by other microbes could enhance *M. restricta* skin colonization and possibly that of other lipid-dependent microbes.

Recent studies have also highlighted FFAs for their ability to modify bacterial sensitivity to antibiotics. For example, the polyunsaturated fatty acid linoleic acid, which is abundant in the stratum corneum, was recently shown to synergize with the cephalosporin cefazolin in the treatment of *S. aureus* biofilms (78). Similarly, the monounsaturated fatty acid palmitoleic acid, which is abundant in human sebum, synergizes with the last-resort antibiotic vancomycin in the treatment of *S. aureus* and was even found to resensitize vancomycin-resistant strains to treatment (79, 80). It remains an open question how, in the specific context of the skin, liberated FFAs might sensitize the microbiota or opportunistic pathogens to antibiotics or host defenses like antimicrobial peptides (AMPs).

In addition to shaping microbial communities, released FFAs can be directly sensed by host cells, leading to downstream effects on skin physiology and immunology. For instance, oleic acid generated during sebum lipolysis has been shown to influence keratinocyte differentiation and compromise skin barrier integrity in a murine model (81). In the context of dandruff and seborrheic dermatitis, *M. globosa*-mediated release of oleic acid is thought to drive scalp irritation, possibly by inducing elevated expression of the pro-inflammatory cytokine IL-36γ (71, 82). Microbial lipolysis of sebum triglycerides may also impact host AMP expression, as FFAs such as oleic acid increase the sebocyte expression of human β-defensin 2 in culture (hBD-2) (7).

Beyond their direct effects, FFAs can also be further processed by host cells into a wide range of bioactive lipid mediators, including eicosanoids, mono- and poly-hydroxylated FFAs, and leukotrienes, which regulate inflammation, immune responses, tissue repair, and regeneration. The host-derived metabolism of skin FFAs into such signaling molecules has been comprehensively reviewed elsewhere (83). In contrast, it is less clear how members of the skin microbiota similarly transform host lipids or FFAs into secondary bioactive molecules with signaling, immunomodulatory, or antimicrobial functions. Defining the extent and specificity of microbial lipid modification on skin represents an important gap in our understanding of host-microbiome interactions.

## MICROBIOTA-TRANSFORMED LIPIDS ON SKIN

Study of the human gut microbiome indicates that the microbiota generates secondary bioactive lipids by transforming exogenous lipids from the host or their diet. The best characterized example is microbiome transformation of primary bile acids to secondary bile acids, which has been recently reviewed in detail in previous studies (84, 85). Importantly, these bioactive lipids can impact host metabolism, the immune response, microbiome composition, and intermicrobial interactions (86–88), highlighting the far-reaching consequences of microbiome-derived bioactive lipids. There is a growing body of literature investigating the transformation of dietary unsaturated fatty acids into bioactive molecules. Gut-colonizing *Bifidobacterium* and *Lactobacillus* species transform dietary linoleic acid into various bioactive hydroxy fatty acid isomers, which have systemic anti-inflammatory and metabolism-altering impacts on the host (89–92). Despite the abundance of lipids and polyunsaturated fatty acids on the skin, there has been limited investigation into the transformation of primary skin lipids by the cutaneous microbiome into secondary bioactive compounds.

Unsaturated fatty acids can be transformed into bioactive hydroxy fatty acids when a double bond is hydrated to form a hydroxyl group. The gut colonizer *Blautia producta* encodes an oleate hydratase (*ohyA*) that converts the host monounsaturated fatty acid oleic acid to an immunomodulatory 10-hydroxystearic acid that suppresses IFN-γ production by CD4^+^ T cells (93). A similar enzyme has been characterized in the transient skin colonizer and major skin pathogen *S. aureus*, where OhyA can generate 10-hydroxystearic acid from oleic acid and 10-hydroxypalmitic acid from palmitoleic acid *in vitro* and in human serum (94). While this is thought to act as a primary defense mechanism to protect the bacteria from the toxicity of unsaturated fatty acids, the produced hydroxy fatty acids also appear to have bioactivity in their ability to modulate the host immune response during *S. aureus* infection (95). While *S. aureus* only transiently colonizes most skin sites apart from the nostrils, related coagulase-negative staphylococci are abundant and stable colonizers of human skin (96). The major skin commensals *S. epidermidis* and *Staphylococcus hominis* encode homologs of *S. aureus* OhyA with 75%–80% amino acid sequence identity. Additionally, within the metatranscriptomic data set produced by Chia et al., oleate hydratase (COG4716) transcripts, although not definitively from staphylococci, are detected on human skin swabs (35). Therefore, skin resident staphylococci, and potentially other skin resident bacteria, are likely equipped to transform sebum or epidermal free fatty acids, such as linoleic, oleic, and palmitoleic acid, into hydroxylated free fatty acids. Notably, the abundant unsaturated skin fatty acid sapienic acid appeared to evade *S. aureus* OhyA activity, as the predicted product 7-hydroxypalmitic acid could not be detected when grown in the presence of sapienic acid (94). Future research should focus on the detection of hydroxy fatty acids on human skin and investigate the contribution of bacterial hydratases in the generation of skin hydroxy fatty acids and their potential effects on the skin’s immune environment.

Skin resident fungi are also equipped to transform skin lipids into bioactive compounds. *Malassezia* yeasts are the most abundant fungal colonizers of healthy human skin, and due to their inability to synthesize fatty acids, they rely entirely on skin lipids for growth. *In vitro* lipidomics on *Malassezia* cells from five species identified over 400 lipidic compounds, including uncommon, bioactive species such as fatty acid esters of hydroxyl fatty acids (97). Since the 1970s, it has been established that *Malassezia furfur* produces dicarboxylic acids as products of unsaturated fatty acid oxidation, including the production of azelaic acid from oleic acid (98, 99). The reported bioactivities of azelaic acid have multiplied since it was first proposed to inhibit melanocytes in the *Malassezia*-associated skin disorder pityriasis versicolor (100). It is now broadly used in dermatology for its antioxidant (99) and anti-inflammatory properties (101). A more recent investigation of skin oxylipins, bioactive oxygenated lipids also known as eicosanoids, detected eight oxylipin species from facial swabs and found that their presence correlated with the presence of different *Malassezia* species. For example, *M. furfur* was positively correlated with the presence of the linoleic acid-derived monohydroxy fatty acid 9-hydroxyoctadecadienoic acid (9-HODE) (102). While the correlation does not reveal if *M. furfur* directly produces the 9-HODE detected on adult human skin, *in vitro* lipidomics detected 9-HODE and other skin oxylipins in *M. furfur* monoculture *in vitro*, thereby suggesting that *Malassezia* is metabolically capable of oxylipin synthesis. If 9-HODE and the related metabolite 13-HODE (13-hydroxyoctadecadienoic acid) are produced by *M. furfur* on skin, they could directly signal to host cells, promoting and inhibiting inflammation, respectively (103–105). As methods for genetic manipulation of *Malassezia* continue to improve, it will be vital to identify the enzymes responsible for oxylipin synthesis, such as lipoxygenases or cytochrome P450s, to decipher their contributions to this skin lipid landscape and skin health.

The bio-transformed skin lipids produced by resident microbes can also act as mediators, shaping intermicrobial interactions. Azelaic acid produced by *M. furfur* displays antibacterial activity *in vitro* against *S. epidermidis* and *C. acnes* (106). *Malassezia sympodialis* was recently shown to generate the hydroxy fatty acid 10-hydroxypalmitic acid (10-HP), which rapidly kills *S. aureus in vitro* and reduces colonization on human skin explants (9). Notably, 10-HP toxicity is dependent on an acidic environment, such as that of human skin, showing limited toxicity in standard laboratory conditions at neutral pH. The production of 10-HP could occur through enzymatic hydration of the n-7 double bond of palmitoleic acid; however, a putative hydratase has not been identified to date. A 1960s study of *M. furfur*, then called *Pityrosporum ovale*, detected the hydroxy fatty acids 9-hydroxypalmitic acid (9-HP) and 9-hydroxystearic acid when the yeast was grown with the saturated fatty acids palmitic acid or stearic acid, respectively (107). This suggests a potential enzymatic mechanism capable of introducing a hydroxyl group within a saturated acyl chain. Like 10-HP, but in contrast to the plant metabolite 16-hydroxypalmitic acid, exposure to 9-HP in acidic conditions rapidly kills *S. aureus in vitro,* indicating a shared antimicrobial mechanism for yeast-derived hydroxy fatty acids (9). Neither 10-HP nor 9-HP have yet been detected on human skin, and their potential impacts on other members of the skin microbiome remain to be investigated as do potential impacts of these yeast-generated hydroxy fatty acids on the local immune environment.

Beyond identifying microbial derivatives of host lipids, it remains an ongoing challenge to characterize their potential bioactivities on the skin. For example, a recent analysis of the scalp metabolome detected fatty acid ethyl esters that are presumably the result of microbial metabolism of sebum fatty acids (108). However, it is unclear if these compounds have an impact on neighboring microbes or the host. Similarly, cholesterol esters are abundant in sebum and can be formed by *S. aureus,* and potentially other staphylococci, as a fatty acid detoxification mechanism, but their bioactivities are not well defined (109). Thus, the functional relevance of many microbially transformed lipid metabolites on the skin remains largely unknown.

## MICROBIOTA-GENERATED LIPIDS ON SKIN

In addition to transforming host lipids on the skin, the cutaneous microbiome generates its own lipids that become part of the skin lipid landscape. For example, skin-resident *Corynebacterium* produces mycolic acid as part of its cell envelope, and this compound contributes to *Corynebacterium accolens*-driven inflammation on the skin of mice fed a high-fat diet (110). The abundant bacterial phospholipid phosphatidylglycerol (PG) is presented by skin-resident Langerhans cells and induces a Th2 response in both peripheral and skin-resident T cells (111). Lysyl PG, a cationic phospholipid produced mainly by gram-positive bacteria, can comprise up to 20%–40% of the phospholipids in *S. aureus* membranes and may directly contribute to allergic immune responses in the skin of individuals with atopic dermatitis (AD), a condition characterized by high levels of *S. aureus* colonization (111, 112). The gram-positive glycolipid lipoteichoic acid (LTA) is a TLR2 ligand and its detection on AD skin, at amounts up to 9.8 µg/mL, correlates with *S. aureus* abundance and disease severity (113, 114). Skin commensal staphylococci also produce LTA as part of their cell envelope, and it has been reported to have roles in suppressing TLR3 inflammation during skin wound repair (115) and increasing skin mast cell expression of the AMP cathelicidin (116). Together, these examples illustrate that primary microbial lipids act as bioactive immunomodulators that can both promote or suppress skin inflammation.

Short-chain fatty acids (SCFAs) are fatty acids with fewer than six carbons that, although volatile and water-soluble, are considered bioactive lipids for the purposes of this review. SCFAs are produced as byproducts of microbial fermentation and include the common SCFAs propionic acid, acetic acid, butyric acid, and valeric acid, among others. On the skin, SCFA production has been best characterized by the lipophilic, aerotolerant anaerobe *C. acnes*, previously named *Propionibacterium acnes* for its production of the SCFA propionate (117, 118). The production of SCFAs on the skin likely results from the fermentation of glycerol in low oxygen or hypoxic niches, such as hair follicles (119, 120). *C. acnes* densely colonizes hair follicles and, as described above, produces lipases that hydrolyze triglycerides, generating both glycerol and fatty acids (69).

Exposure to SCFAs can directly modulate host cells. *C. acnes* production of the SCFA propionate has been observed to stimulate keratinocyte lipid synthesis and increase triglyceride content in human cell monolayers and constructed epidermidis models, as well as *in vivo* on murine skin (121). These effects of propionate on keratinocytes are dependent on the receptor PPARα (peroxisome proliferator-activated receptor) and enhance multiple aspects of the skin barrier, including increased hydration and antimicrobial activity (121). Propionate and valerate produced by *C. acnes* can also influence keratinocytes by inhibiting histone deacetylase activity, thereby inducing these normally immune-tolerant cells to produce proinflammatory cytokines in response to microbial antigens (120). In addition to keratinocytes, *C. acnes*-produced propionate drives proinflammatory responses in sebocytes by inhibiting histone deacetylase activity and signaling directly through the G protein-coupled receptors FFAR2, FFAR3, and HCAR2 (122). These findings demonstrate that SCFAs produced by the skin microbiome, particularly by *C. acnes*, play multifaceted roles in regulating skin barrier function and immune responses.

Microbial produced SCFAs also impact other members of the skin microbiome and invading pathogens. *C. acnes-*produced propionate inhibits *S. aureus* growth *in vitro* and reduces *S. aureus* colonization and lesion size in a murine model of cutaneous infection (123). *C. acnes* supernatants, containing SCFAs, also inhibit *S. aureus* and, to a greater extent, *S. epidermidis* biofilm formation *in vitro* (119). Specifically, the *C. acnes* SCFAs propionate, isobutyrate, and isovalerate act by inhibiting *S. epidermidis* polysaccharide synthesis, which is essential for biofilm formation, and, through weakening of the biofilm, increase *S. epidermidis* sensitivity to the antibiotics ampicillin and doxycycline (119). While *C. acnes* is a major producer of SCFAs on skin, other skin resident bacteria can generate SCFAs *in vitro*, as has been shown for staphylococci such as *S. hominis*, *S. epidermidis*, and *S. warneri*, which produce isovaleric acid and acetic acid (121). Acetic acid, a two-carbon SCFA, can alter chromatin organization and histone acetylation in *M. restricta* and *M. sympodialis*, leading to strong repression of fungal transcription (124). The acidification caused by staphylococcus-produced acetic acid may also alter *M. restricta* sensitivity to antifungals by altering ergosterol levels in the cell membrane (125). Overall, microbially derived SCFAs are poised to play an influential role in regulating microbial interactions and community dynamics within the skin ecosystem.

As technologies and analytical methods for the capture, detection, and annotation of skin lipids continue to advance, it is likely that additional microbially derived lipids with bioactive properties will be identified. Some of these lipids may also provide templates for chemical engineering to enhance desirable functions, such as a butyrate derivative with increased antimicrobial activity against *S. aureus* (126). Notably, a recently developed workflow for human sebum analysis revealed unusual straight-chain polyunsaturated fatty acids and branched-chain saturated fatty acids of unknown origin, but that may originate from the microbiota (127). Looking forward, key challenges include linking specific bioactive lipids to their microbial producers and characterizing their bioactive roles within the native skin environment. Approaches and technologies to address these challenges are described in Table 1. These include applying integrated multi-omics approaches and imaging mass spectrometry to correlate microbial presence with metabolite abundance and location *in situ* (128, 129), generating mutant microbial strains to link gene and protein function with metabolite presence using mono-colonized animal models (130, 131), and stable isotope probing and Raman spectroscopy to identify specific compounds in metabolically active cells (132–136). Given the complexity of the skin lipid landscape, it is likely that a combination of these approaches will be necessary to definitively link specific microbes with the production of bioactive lipids.

**TABLE 1:** Methods to dissect microbiome-lipid relationships on the skin^*a*^

Approach/technology	Strengths	Limitations	References
Integrated multi-omics – Metagenomics and metabolomics – Targeting or untargeted metabolite identification – Identify metabolite-microbe pairs	– Unbiased, culture-independent – *In situ* sampling – Hypothesis generating	– Correlation-based – Does not distinguish host versus microbial production	(57, 130)
Imaging mass spectrometry – Coupled with microscopy – Resolve spatial relationships between metabolites and microbes	– Can be performed directly on skin samples – Retains spatial information	– Correlation-based – Does not distinguish host versus microbial production	(128, 129)
Gene-metabolite analysis – Compare metabolite production by wild-type and mutant strains	– Able to test definitive metabolite-producer relationships – Can be assessed *in vitro* or in animal models	– Culture-dependent – Requires genetic tractability – Unlikely to be used *in situ* on human skin	(72, 93)
Gnotobiotic animal models – Mono-colonization or defined community colonization of germ-free animals coupled with quantitative metabolomics	– Links metabolite presence with specific microbe(s) – Strengthened by use with genetically altered strains	– Germ-free mice have altered skin phenotypes – Culture-dependent – Does not distinguish host versus microbial production	(130, 131)
Stable isotope probing – Tracing the incorporation of isotopically labeled compounds into new microbial metabolites – Often combined with mass spectrometry or Raman spectroscopy	– Culture-independent – Can be coupled with Raman spectroscopy, imaging mass spec., fluorescence *in situ* hybridization – Can incorporate spatial information with single-cell resolution	– Limited detection of transformed host metabolites – Can be difficult to differentiate producers from microbial cross-feeding	(133, 134)
Surface-enhanced Raman spectroscopy – Quantitative metabolomics within single microbial cells – Raman scattering–two-photon fluorescence *in situ* hybridization (SRS-FISH) combines metabolic fingerprinting with molecular identification	– Label-free, non-destructive – Single-cell resolution – Can be coupled with stable isotope probing and various imaging approaches – Can detect intracellular and extracellular metabolites	– Background signal can obscure spectra in biological samples – Substrate/nanoparticle selection may need to be optimized for specific metabolite detection	(135, 136)

^*a*^ Example experimental approaches and technologies available to characterize direct connections between members of the cutaneous microbiome and their bioactive lipid metabolites, particularly *in situ*.

## MICROBIAL LIPID METABOLISM IN SKIN DISEASE

Epidermal and skin surface lipids like sebum are central components of the skin barrier; hence, it is not surprising that many skin diseases, both rare and common, are associated with alterations in lipid composition (137). These changes in host lipid composition and metabolism in skin diseases like AD and psoriasis have been reviewed elsewhere (138, 139). It is, however, less clear how the skin microbiome influences disease-associated lipid changes or how microbially derived lipids, whether generated *de novo* or transformed from the skin, influence disease state. Some of the best-characterized examples to date concern acne vulgaris, an inflammatory disease of the pilosebaceous unit tightly linked to sebum production (140).

Acne vulgaris has long been associated with *C. acnes*, despite this species residing within the hair follicles, or pilosebaceous units, of both healthy and acne skin (141). Metagenomics has since revealed an array of *C. acnes* phylotypes, with some associated with healthy skin and others with acne. Genomic comparison between these phylotypes has produced several plausible models of *C. acnes-*driven disease (142). One model proposes that phylotypes associated with healthy skin have reduced lipase activity, releasing fewer inflammatory FFAs that contribute to inflammation in acne (142, 143). Indeed, the non-acne *C. acnes* type II strains have potential loss of function mutations expected to impact two tandemly encoded triacylglycerol lipases (142). Recent analysis of the *C. acnes* lipidomes from *in vitro* grown cultures indicate that different phylotypes vary in their lipid composition; however, only a single strain was used to represent each phylogroup. Despite this limitation, the study reveals intriguing differences that could impact host interactions, such as the production of sphingomyelin 35:1 by phylotypes associated with healthy skin (144). Other *C. acnes*-derived lipids are also poised to exacerbate inflammation in acne. As mentioned above, the bacterial glycolipid LTA is a TLR2 ligand (114), and TLR2 activation by *C. acnes* has been reported to occur in acne (145). SCFAs produced by *C. acnes* may also contribute to inflammation in acne by altering histone modifications, which regulate the expression of proinflammatory cytokines by keratinocytes (120). Oxidation of the abundant sebum lipid squalene may also contribute to the pathogenesis of acne vulgaris, and *C. acnes* may have an active role in the generation of such lipid peroxides (146). In *in vitro* models, squalene peroxides are comedogenic, having the ability to lead to occlusion of skin pores, and can induce inflammation (147–150). One possible mechanism by which *C. acnes* may contribute indirectly to the production of squalene peroxides is through the interaction of its secreted porphyrins with oxygen and ultraviolet (UV) radiation (146). Squalene peroxides may also have a role in fungal acne, or *Malassezia folliculitis*, which is often misdiagnosed as acne vulgaris but instead is associated with *Malassezia-*driven inflammation of the pilosebaceous unit (151). The squalene peroxides, squalene monohydroperoxide and malondialdehyde, were recently detected *in vitro* in cultures of *M. restricta*-provided squalene and linoleic acid (152). While studies have not yet associated this lipid peroxide with fungal acne or demonstrated its production *in situ* by *Malassezia*, it has been proposed to play a possible role in *Malassezia-*associated dandruff.

*Malassezia* species have been implicated in dandruff, a flaking disorder of the scalp, since the late 1800s. Seborrheic dermatitis is a more severe *Malassezia*-linked disorder, characterized by chronic inflammation of the scalp, which may spread to the face and other sebaceous sites (153). As stated above, *Malassezia* secretes numerous lipases to hydrolyze lipids in the skin environment and liberate FFAs. One model for *Malassezia*’s involvement in dandruff and seborrheic dermatitis is that this lipase activity releases unsaturated fatty acids, such as oleic acid, that are not consumed by the yeast. These FFAs may act as irritants on the skin, causing scaling and flaking in susceptible individuals (71, 154); however, the evidence remains inconclusive (155). Recently, it has been proposed that squalene peroxides produced by *Malassezia* contribute to dandruff and seborrheic dermatitis, as these oxidized lipids are present in higher amounts on affected scalps (152, 156). Application of squalene peroxides to *in vitro* epidermal models revealed an impact on skin barrier organization and permeability, providing additional support to the hypothesis that these lipid metabolites may affect keratinocytes in dandruff and seborrheic dermatitis (152). Given that squalene is a potent antioxidant, the specific oxidizing factors on human skin responsible for peroxide generation, including potential contributions from *Malassezia* and the cutaneous microbiome, have yet to be fully elucidated.

Atopic dermatitis (AD; also called eczema) is a highly prevalent, chronic skin disorder characterized by altered skin lipid composition and microbiome dysbiosis (157). A hallmark of many AD lesions is abundant colonization by *S. aureus*, and significant research has focused on elucidating the role of *S. aureus* in AD onset and progression (112, 158). A current model suggests that host and environmental factors result in disrupted organization of the stratum corneum and a compromised barrier that facilitates *S. aureus* colonization; the bacteria then employ numerous virulence factors that enhance colonization and contribute to the progression of the disease (158). The role of microbially derived lipids in AD has not been thoroughly investigated. There is evidence that *S. aureus* LTA and Lysyl PG, which can be detected on AD skin, induce inflammation, and LTA specifically has been found to cause hyperproliferation of keratinocytes in mice after intradermal injection (111, 159). A hallmark of epidermal disruption in AD lesions is the reduction of the barrier proteins filaggrin and loricrin (160). Injection of LTA into the mouse dermis induces neutrophil recruitment that coincides with reduced filaggrin and loricrin expression, indicating significant barrier damage similar to AD (159). Separate from *S. aureus* colonization, there is growing evidence that SCFA production by the gut microbiome improves the skin barrier in AD murine models (161, 162). Given the changes in the skin microbiome during AD and the discovery that microbially derived antimicrobial fatty acids can kill *S. aureus* (9), it is likely that new roles for microbially derived lipids will be characterized, potentially aiding in the treatment of AD.

The contributions of microbially derived lipids to skin disease discussed above are primarily in conditions with a strong link or association with a specific microbe or to well-defined changes in the skin microbiome. However, many other skin diseases exhibit alterations in the lipid landscape; yet, the role of the microbiome in these conditions has not been thoroughly investigated. As our understanding of how the microbiome changes in response to and contributes to diseases, such as ichthyosis and psoriasis, improves, we may uncover valuable insights into how microbial lipids influence disease pathogenesis. For instance, exploring the contribution of microbial lipids in the progression of the chronic autoinflammatory disorder Hidradenitis Suppurativa (HS) may unlock new understandings of the disease. HS is characterized by recurrent skin abscesses that can have bacterial involvement (163). Recent lipidomic analysis of HS lesions detected unique lipid profiles compared to healthy skin (164, 165), with changes in oxylipin lipid mediators (165), and identification of triglycerides with odd-chain fatty acids that are hypothesized to originate from lesional bacteria, the main producers of odd-chain fatty acids (166). While it remains to be determined whether these odd-chain fatty acids are of microbial origin and whether they contribute to HS pathophysiology, the convergence of microbial and lipidomic alterations in HS makes this pathology an ideal candidate for the investigation of the role of microbial lipids in disease (165).

## CONCLUSIONS

The lipid composition of human skin is closely linked to both shifts in the resident microbiota and the development of skin disease. However, the full extent to which the skin microbiome actively shapes the cutaneous lipid landscape through the generation, modification, or biotransformation of bioactive lipids remains underappreciated. Rather than acting independently, skin lipids and resident microbes exist in a dynamic and reciprocal relationship in which each continually influences the other. Recognizing the constituents of the microbiome as active participants in shaping skin lipid composition will be essential for advancing our understanding of skin health and disease and for identifying new therapeutic opportunities.

The skin microbiome and its metabolites are being actively explored in therapeutic contexts, especially via live microbial transplantation for AD; however, as is common in early-stage therapeutic development, outcomes have been variable (167–171). An appealing aspect of leveraging microbially derived lipids for the treatment of skin disease is the flexibility of therapeutic approaches. These strategies may include a prebiotic approach, in which topical application of host lipid precursors is used to shape microbial metabolism; a probiotic approach, involving transplantation of lipid-producing or transforming microbes; or direct application of the purified lipid compounds themselves. In addition to their application as therapeutics, microbially derived skin lipids have the potential to serve as biomarkers for health or disease. Sebum is already being explored as a non-invasive source of biomolecular data for several non-dermatological diseases, including neurological and metabolic disorders such as Parkinson’s disease (PD) and type 2 diabetes, respectively (172–174). While further research is necessary, a small study investigating volatile compounds associated with PD identified that the long-chain alkane eicosane, previously shown to be produced by *Actinobacteria*, was enriched in people with PD (65, 175). Together, these findings highlight the need to further investigate microbially derived skin lipids as both therapeutic agents and biomarkers of health and disease.

While there is strong rationale to investigate microbial skin lipids and their bioactivities, there are technical hurdles that must be overcome. First, untargeted metabolomics investigations in combination with improved annotation to capture and identify unusual and low-abundance microbial lipid species are necessary. The next challenge is associating the presence of a candidate lipid species with a specific microbial species. For these studies, it is often necessary to culture candidate species in the laboratory, which is not yet possible for all skin-resident microbes (176, 177). Furthermore, the laboratory culture conditions need to recapitulate the skin environment where the metabolism of candidate lipids is predicted to take place. By employing recent recipes for skin-like media and synthetic sebum formulations, culture conditions may become less of an obstacle moving forward (38, 178). Finally, the bioactivity of each microbial lipid needs to be investigated, and particularly for immune modulation or skin barrier effects, *in vivo* models that recapitulate human skin in health and disease are needed. If a host lipid precursor is necessary for microbial generation of a bioactive lipid and that precursor is not present in mice, then current *in vivo* models may not be suitable for the investigation. In this example, it may be appropriate to leverage *ex vivo* models such as human skin organoids. By addressing these challenges and identifying microbially derived lipids, characterizing their bioactivities, and linking them to specific microbial producers, we can uncover new mechanisms of barrier function, immunity, and microbial homeostasis, laying the groundwork for novel therapeutic and diagnostic approaches.
